# Structure Elucidation and Biosynthesis of Orobanchol

**DOI:** 10.3389/fpls.2022.835160

**Published:** 2022-02-09

**Authors:** Takatoshi Wakabayashi, Kotomi Ueno, Yukihiro Sugimoto

**Affiliations:** ^1^Graduate School of Agricultural Science, Kobe University, Kobe, Japan; ^2^Faculty of Agriculture, Tottori University, Tottori, Japan

**Keywords:** cytochrome P450 monooxygenase, germination, root parasitic weeds, stereochemistry, strigolactone

## Abstract

Strigolactones (SLs), a class of phytohormones that regulate diverse developmental processes, were initially characterized as host-derived germination stimulants for seeds belonging to the genera *Striga*, *Orobanche*, and *Phelipanche*. Orobanchol (**1**), which is detected in the root exudates of several plants and recognized as a prevalent SL, was first isolated from the root exudates of red clover as a germination stimulant for *Orobanche minor* in 1998. However, the structure of this stimulant proposed at that time was disputable considering its predicted germination-inducing activity for *Striga gesnerioides*. The genuine structure of orobanchol was elucidated following a decade-long controversy, which ultimately facilitated the understanding of the importance of SL stereochemistry in *Striga* seed germination. Recently, studies focusing on clarifying the biosynthesis pathway of orobanchol are being conducted. Cytochrome P450 monooxygenases are involved in orobanchol biosynthesis downstream of carlactonoic acid (CLA) via two pathways: either through 4-deoxyorobanchol or direct conversion from CLA. Substantial progress in the identification of more SL structures and clarification of their biosynthetic mechanisms will further contribute in the comprehension of their structural diversity’s functional importance and agricultural applications. Herein, we have reviewed the history leading to the discovery of the genuine structure of orobanchol and the current understanding of its biosynthetic mechanisms.

## Introduction

**FIGURE 1:**
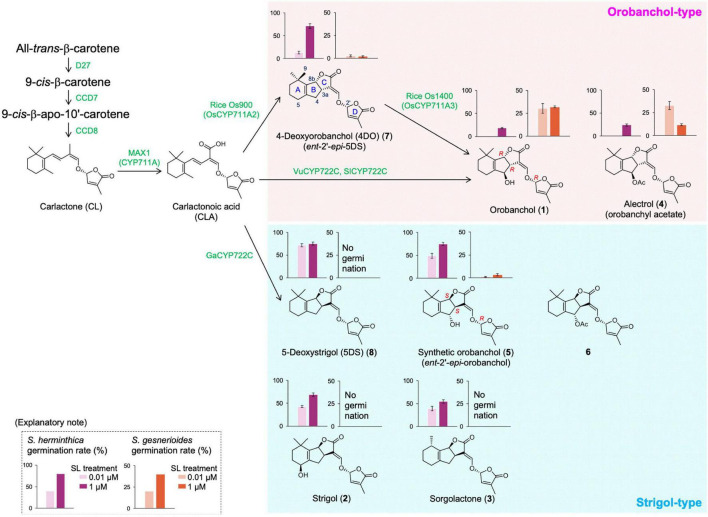
Proposed strigolactone (SL) biosynthesis pathway from β-carotene and the seed germination induction of *Striga* by each SL. The sequential reactions catalyzed by D27, CCD7, and CCD8 enzymes produce carlactone (CL) from all-*trans*-β-carotene. CL is further converted to carlactonoic acid (CLA) by the CYP711A subfamily. Downstream of CLA, Os900/OsCYP711A2 in rice (*Oryza sativa*), VuCYP722C and SlCYP722C in cowpea (*Vigna unguiculata*) and tomato (*Solanum lycopersicum*), respectively, and GaCYP722C in cotton (*Gossypium arboreum*) can produce 4-deoxyorobanchol, orobanchol, and 5-deoxystrigol, respectively. The bar graphs at the top of each SL structure indicate the germination rates of *S. hermonthica* and *S. gesnerioides* induced by each SL as reported previously (Ueno et al., 2011b; Nomura et al., 2013).

Strigolactones (SLs) were initially characterized as germination stimulants for seeds belonging to the genera *Striga*, *Orobanche*, and *Phelipanche*, which are a renowned group of root parasitic weeds of global economic importance (Parker, 2009). Strigol (**2**), the first canonical SL structurally defined, was isolated from the root exudates of cotton (*Gossypium hirsutum*) (Cook et al., 1966, 1972). Following the isolation of strigol, the SLs sorgolactone (**3**) (Hauck et al., 1992), alectrol (**4**) (Müller et al., 1992), and orobanchol (**1**) (Yokota et al., 1998) were isolated from the root exudates of sorghum (*Sorghum bicolor*), cowpea (*Vigna unguiculata*), and red clover (*Trifolium pratense*), respectively. Consequent studies revealed that SLs not only promoted hyphal branching of arbuscular mycorrhizal fungi (Akiyama et al., 2005) but also represented a new class of phytohormones that regulated plant architecture (Gomez-Roldan et al., 2008; Umehara et al., 2008). Structurally, canonical SLs consist of tricyclic lactone (ABC ring) and butenolide (D ring) connected with an enol ether bridge (Figure 1). The structures of strigol (**2**) and sorgolactone (**3**) were unambiguously determined by X-ray crystallographic analysis and organic synthesis (Brooks et al., 1985; Sugimoto et al., 1998), whereas the genuine structures of orobanchol (**1**) and alectrol (**4**) were eventually established in 2011 (Ueno et al., 2011b). Orobanchol has been detected in the root exudates of numerous plants, including Fabaceae, Solanaceae, a few Gymnosperm species, and rice (*Oryza sativa*) (Xie, 2016; Wang and Bouwmeester, 2018). Several derivatives of orobanchol, such as its acetate, orobanchyl acetate (alectrol), fabacol that contains an epoxide group, and solanacol that has an aromatic A-ring, have also been identified (Müller et al., 1992; Xie et al., 2007, 2009). The illustration of the genuine structure of orobanchol allowed canonical SLs to be divided into two subgroups that were categorized in terms of their C-ring configuration, the orobanchol- and strigol-types. The C-ring configuration was found to be essential in fulfilling the structural requirements of the canonical SLs for inducing germination in *Striga gesnerioides* seeds (Ueno et al., 2011a; Nomura et al., 2013). The classification of the canonical SLs into the two subgroups presented an avenue to study the enzymes involved in their biosynthesis from the common intermediate, carlactonoic acid (CLA) (Zhang et al., 2014; Wakabayashi et al., 2019, 2020; Mori et al., 2020).

This review outlines the course of determining the genuine structure of orobanchol, its biological importance as a seed germination stimulant for the genus *Striga*, and its biosynthesis pathway at biochemical and molecular levels. The components involved in the biosynthesis of orobanchol and its related canonical SLs that are yet to be clarified are also discussed.

## Structure and Germination-Inducing Activity

### History Leading to the Determination of the Genuine Structure of Orobanchol

Orobanchol was isolated from the root exudates of red clover as the first germination stimulant for *Orobanche minor*, together with alectrol (Yokota et al., 1998). Alectrol had been previously isolated from the root exudates of cowpea as an isomer of strigol and a germination stimulant for *Alectra vogelii* and *S. gesnerioides*. A structure for alectrol was proposed based on a detailed comparison of its spectroscopic data with those of strigol (Müller et al., 1992). Since the isolated amount of orobanchol from red clover was constrained, it was considered to be a strigol-related compound and no specific structure for it was proposed. Following these reports, a series of strigol analogs, including the tentative structures of orobanchol and alectrol, were synthesized (Matsui et al., 1999a,b). The structure **5** was assigned to orobanchol by comparing its ^1^H NMR spectra and chromatographic behavior in gas chromatography–mass spectrometry, in which the C-ring configuration was consistent with that of strigol (**2**) (Figure 1). Chiroptical data were not utilized in the structural determination process. After about a decade, alectrol was independently re-isolated from the root exudates of red clover and cowpea (Matsuura et al., 2008; Xie et al., 2008), and its structure was reported as an acetylated product of synthetic orobanchol (**6**). However, synthetic orobanchol (**5**) and its acetate (**6**) did not induce seed germination in *S. gesnerioides* (Ueno et al., 2011a), indicating that the assigned structures of these SLs were controversial. These results triggered the re-isolation of the germination stimulants of *S. gesnerioides* from the root exudates of cowpea and red clover (Ueno et al., 2011b). The details of the bioassay-guided re-isolation and unambiguous structural elucidation of these stimulants have been described in a previous review (Ueno et al., 2015). In brief, two stimulants were isolated from both cowpea and red clover root exudates. The ^1^H NMR spectra of these stimulants suggested that they were canonical SLs having an oxygen functional group at C-4 in the B-ring. The chromatographic behavior of the stimulants in liquid chromatography–tandem mass spectrometry (LC-MS/MS) analysis was inconsistent with that of synthetic orobanchol (**5**) and its acetate (**6**) but consistent with their respective 2′-epimers. Additionally, the circular dichroism spectra of the stimulants were vertically inverted compared with the 2′-epimers of **5** and **6**. Therefore, the absolute structures of orobanchol and alectrol were determined to be **1** and **4**, respectively (Figure 1).

Figure 1 (**1**) illustrates the genuine structure of orobanchol, which has the (3a*R*, 8b*R*, 2′*R*)-configuration. Contrary to strigol (**2**), orobanchol (**1**) demonstrates an inverted BC-junction configuration. Dehydroxylated orobanchol and strigol, 4-deoxyorobanchol (4DO) (**7**) and 5-deoxystrigol (5DS) (**8**), respectively, have opposite C-ring configurations, and hence, 5DS is also known as *ent*-2′-*epi*-4DO. It was predictable that the absolute skeletal configuration of redefined orobanchol was the *ent*-2′-*epi*-form of the strigol skeleton. Before the structural revision, 2′-*epi*-5-deoxystrigol (*epi*-5DS) had been found in the hydroponic culture media of rice seedlings (cv. Shiokari) by LC-MS/MS analysis using a reversed-phase octadecyl silica (ODS) column (Umehara et al., 2008). The detected “*epi*-5DS” is presumed to be 4DO (*ent*-2′-*epi*-5DS, **7**), since an LC-MS/MS analysis with an ODS column only distinguishes between diastereomers. Subsequently, it was reported that rice produces orobanchol in addition to *epi*-5DS (Jamil et al., 2011). Moreover, the absolute configuration of fabacyl acetate isolated from pea (*Pisum sativum*) was the same as that of 4DO (Xie et al., 2009). Therefore, the correction of the absolute configuration of orobanchol was readily accepted by the community of SL researchers.

### Importance of the Stereochemistry of Orobanchol in Inducing Seed Germination

The structure of both synthetic (**5**) and naturally occurring orobanchol (**1**) have the *R*-configuration at C-2′, which is an important structural feature for shoot branching inhibitory activity. Synthetic (**5**) as well as naturally occurring orobanchol (**1**) has shown to inhibit shoot branching in rice (Umehara et al., 2015). In contrast, during the structural examination of orobanchol, the importance of its stereochemistry in inducing seed germination in *S. gesnerioides* was suggested (Ueno et al., 2011a; Nomura et al., 2013; Figure 1). Detailed structure–activity relationship studies on 36 SL stereoisomers, including naturally occurring and synthetic ones, exemplified the strict structural requirements of the canonical SLs for inducing germination in *S. gesnerioides* seeds. Only a limited number of compounds, including orobanchol, induced significant germination in *S. gesnerioides* seeds. The SLs with high germination-inducing activity for *S. gesnerioides* seeds have a consistent C-ring configuration with that of orobanchol (**1**) and a hydroxy group at C-4 with β-orientation or at C-9, the *trans* methyl group against the C-ring. Notably, these germination inducers of *S. gesnerioides* induced a lower germination rate in *S. hermonthica*, which had a more sensitive response to synthetic orobanchol (**5**) that has the same configuration as strigol (**1**). Sorghum, one of the host plants of *S. hermonthica*, exudes sorgomol, which also has the same configuration as strigol (**2**). Additionally, SLs with the same C-ring configuration as strigol suppressed the orobanchol-induced germination of *S. gesnerioides* seeds. Therefore, root parasitic weeds may have evolved to germinate closer to the roots of compatible host plants where they can parasitize by strictly recognizing the configuration of the SLs. These findings indicated that not only the total amount but also the composition of SLs exuded by the host plants influence the adverse effects caused by parasitic weeds. Studies focused on elucidating the biosynthesis pathway of orobanchol were consequently pursued.

## Biosynthesis

### Two Distinct Biosynthesis Pathways of Orobanchol

In SL biosynthesis, D27 isomerizes all-*trans*-β-carotene to 9-*cis*-β-carotene, followed by CCD7-induced cleavage to form 9-*cis*-apo-10′-carotenal, and further CCD8 catalyzed conversion to the SL biosynthetic precursor, carlactone (CL) (Alder et al., 2012; Seto et al., 2014; Figure 1). Cytochrome P450 monooxygenase (CYP) AtCYP711A1 encoded by *MORE AXIALLY GROWTH 1* (*MAX1*) converts CL to CLA and is responsible for the branching phenotype observed in Arabidopsis and OsCYP711As, which belong to the same subfamily of rice, also catalyze this reaction (Abe et al., 2014; Zhang et al., 2014). Subsequently, the conversion of CL to CLA has been indicated to be a common function of the CYP711A subfamily in different plant species, suggesting that CLA is also a precursor in SL biosynthesis (Yoneyama et al., 2018). Based on the commonality of planar structure of the basic skeleton, it was assumed that the canonical SLs downstream of CLA first generated the tricyclic skeletons (5DS and 4DO), and then underwent hydroxylation and further modifications to generate strigol, orobanchol, and their acetates.

The pioneering study on canonical SL biosynthesis in japonica rice first elucidated the biosynthesis pathway of orobanchol through the conversion to 4DO (Zhang et al., 2014). The rice CYP711A subfamily shares the common functionality of CL to CLA conversion and is also involved in the conversion to orobanchol. In the rice CYP711A subfamily, OsCYP711A2/Os900 catalyzes the conversion of CL to 4DO via CLA, and OsCYP711A3/Os1400 catalyzes the hydroxylation of 4DO at C-4 to ultimately form orobanchol. Based on these results, it was assumed that the CYP711A subfamily in other plant species is also responsible for the conversion of CLA to the respective canonical SLs, including orobanchol; however, the catalyzing property of this subfamily that converts CL and CLA to canonical SLs in seed plants has been exclusively identified only in rice (Yoneyama et al., 2018). Alternatively, conventional feeding experiments observed that orobanchol producing plants (cowpea, red clover, pea, red bell pepper) that were exogenously administered with 4DO did not convert it to orobanchol, whereas CLA was converted to orobanchol (Iseki et al., 2018; Ueno et al., 2018). These results further suggested a direct biosynthesis pathway of orobanchol from CLA in addition to the indirect pathway through the conversion to 4DO, involving the OsCYP711A2/Os900 and OsCYP711A3/Os1400 of rice. The involvement of other enzymes besides the CYP711A subfamily in canonical SL biosynthesis has been suggested.

### Direct Conversion of Carlactonoic Acid to Orobanchol by CYP722C in Orobanchol Producing Plants (Cowpea and Tomato)

Uniconazole-P, a CYP inhibitor, suppressed the conversion of CLA to orobanchol in cowpea, suggesting that CYP plays a role in this conversion. *VuCYP722C*, whose function was unknown, was highlighted as a candidate gene via gene co-expression analysis using RNA-seq data of cowpea roots grown under various conditions with different SL production levels. The results of the *in vitro* enzyme assay conducted with a crude enzyme of recombinant VuCYP722C demonstrated that the enzyme produced orobanchol and its diastereomer, *ent*-2’-*epi*-orobanchol (**5**), with an opposite configuration in the C-ring, in approximately equal amounts using CLA as a substrate. Additionally, presumed 18-hydroxy-CLA was detected in the enzyme-reaction mixtures. VuCYP722C did not catalyze the conversion of 4DO to orobanchol, which is consistent with the previous results of the feeding experiments (Wakabayashi et al., 2019).

The enzymatic function of SlCYP722C was analyzed in tomato (*Solanum lycopersicum*), another representative orobanchol producer. The changes in *SlCYP722C* gene expressions were similar to that of known SL biosynthetic genes; upregulated under phosphate-deficient conditions that promote SL production. The recombinant enzyme exhibited an activity that was comparable to that of cowpea VuCYP722C. These results further demonstrated the existence of an alternative orobanchol biosynthesis pathway involving CYP722C (Wakabayashi et al., 2019; Figure 1).

### The Function of CYP722C in Tomato, a Model Orobanchol Producing Plant

Analyses of *SlCYP722C* knockout tomato (*SlCYP722C*-KO) plants established the involvement of the CYP722C subfamily in the direct conversion of CLA to orobanchol. The root exudates of *SlCYP722C*-KO plants, wherein the CRISPR/Cas9 system was employed to disrupt the gene by genome editing, orobanchol and solanacol (a possible derivative of orobanchol) were demonstrated to be below-detection level using LC-MS/MS analysis, and instead, CLA accumulation was observed. The modified profiles of the lacking canonical SLs were also reflected in their germination stimulation activities in the seeds of root parasitic weeds. In other words, the root exudates of *SlCYP722C*-KO induced significantly less germination in *S. hermonthica*, *O. crenata*, and *Phelipanche aegyptiaca* seeds than those of wild-type. Interestingly, the *SlCYP722C*-KO plants appeared similar to the wild-type plants and they did not show the prominent phenotypes of an SL-deficient mutant, such as increased shoot branching and reduced stem length (Wakabayashi et al., 2019). These observations depicted that canonical SLs were not essential for regulating shoot branching in tomato plants and further suggested that the branching inhibiting hormone was a non-canonical SL lacking the ABC ring structure derived from CLA, as *MAX1*/*CYP711A* mutation induces increased shoot branching (Zhang et al., 2018; Wakabayashi et al., 2019). Accordingly, canonical SLs could be more important as rhizosphere signaling molecules than shoot branching inhibitors, and preferentially secreted into the soil and facilitate plant–microbe and plant-plant communications.

## Discussion

**FIGURE 2:**
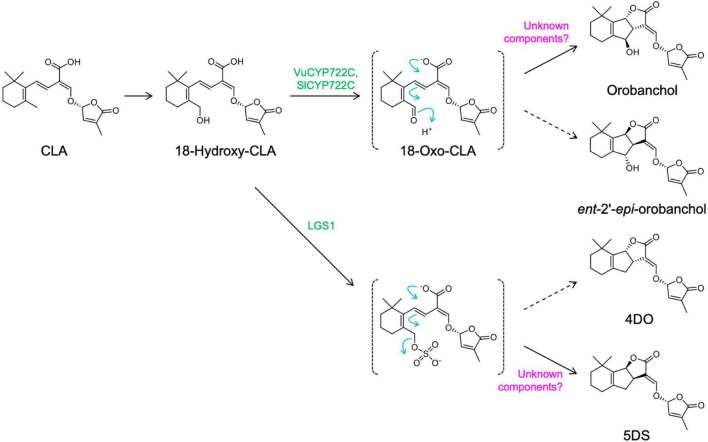
Proposed mechanisms for generating canonical strigolactones with BC ring formation. Additional components may be necessary for the stereospecific conversion of 18-oxo-CLA to orobanchol and the sulfate ester of 18-hydroxy-CLA to 5DS (CLA, carlactonoic acid).

The determination of the genuine structures of orobanchol (**1**) and its acetate, orobanchyl acetate (alectrol) (**4**), has put an end to the long controversy regarding these structures (Ueno et al., 2011b). Orobanchol is also converted to its didehydro derivatives, didehydro-orobanchol isomers, although their structures and enzymes responsible for the conversion remain elusive (Zhang et al., 2018). Identification of CYP722C provided additional information on the biosynthesis pathway of orobanchol from β-carotene at a molecular level. The *in vitro* enzymatic reactions of VuCYP722C and SlCYP722C with CLA as a substrate yielded orobanchol and its diastereomer, *ent*-2’-*epi*-orobanchol, as products (Wakabayashi et al., 2019). These reactions further suggested that the members of the CYP722C subfamily catalyzed the two-step oxidization at the C-18 position in CLA, producing 18-oxo-CLA through 18-hydroxy-CLA. The 18-oxo-CLA then undergoes the BC ring closure reaction, without stereoselective control, to yield orobanchol isomers (Figure 2). Recently, it was reported that in a co-culture system of *Escherichia coli* and *Saccharomyces cerevisiae* co-expressing SL biosynthesis genes, orobanchol is generated by the co-expression of *VuCYP722C* with the upstream SL biosynthesis genes. However, the production of its diastereomer has not been described (Wu et al., 2021). Therefore, a more detailed functional analysis of the CYP722C subfamily is necessary. The formation of the BC ring without the stereoselective control of the C-ring configuration is also found in 5DS biosynthesis involving the LOW GERMINATION STIMULANT 1 (LGS1) of sorghum. It is strongly suggested that LGS1, encoding for the sulfotransferase protein, catalyzes the sulfonation of 18-hydroxy-CLA and provides an easier leaving group to afford a spontaneous non-selective BC ring formation, resulting in simultaneous production of 5DS and 4DO (Yoda et al., 2021; Figure 2). Altogether, there is likely an involvement of unknown components in the stereoselective control of the C-ring in the conversion of 18-oxo-CLA to orobanchol and the sulfate ester of 18-hydroxy-CLA to 5DS.

The CYP722C subfamily is widely conserved in dicot plants, regardless of the type of SL produced (orobanchol- or strigol-type). GaCYP722C of cotton (*G. arboreum*), which generates 5DS as a strigol-type SL, catalyzes the conversion of CLA to 5DS, but it is not involved in the conversion to 4DO (Wakabayashi et al., 2020). Alternatively, GaCYP722C catalyzes stereoselective BC ring formation, unlike VuCYP722C and SlCYP722C. In addition, it has been reported that the CYP722Cs of birdsfoot trefoil (*Lotus japonicus*) and woodland strawberry (*Fragaria vesca*) are involved in the conversion of CLA to 5DS (Mori et al., 2020; Wu et al., 2021). The CYP722C subfamily members are the key enzymes involved in the biosynthesis of canonical SLs, regardless of their C-ring configuration. The differences in their catalytic activity may be due to the differences in the amino acid residues at the catalytic site and conformation of the protein structure domains. Structural biological approaches may clarify the mechanisms regulating the C-ring configuration in canonical SL biosynthesis.

Although much progress has been made in understanding the diverse structures of SLs and their biosynthetic mechanisms, the physiological significance of SL stereochemistry remains largely unexplored. If the mechanism by which plants control the stereochemistry of the C-ring to produce both types of SLs could be elucidated, it would then become possible to artificially control their structures through genetic engineering. The knowledge obtained from this approach will greatly contribute in comprehending the role of SLs. Additionally, the precise control of SL functions is predicted to have agricultural applications, such as management of root parasitic weeds and promotion of mycorrhizal symbiosis.

## Author Contributions

TW, KU, and YS wrote the review. All authors contributed to the article and approved the submitted version.

## Conflict of Interest

The authors declare that the research was conducted in the absence of any commercial or financial relationships that could be construed as a potential conflict of interest.

## Publisher’s Note

All claims expressed in this article are solely those of the authors and do not necessarily represent those of their affiliated organizations, or those of the publisher, the editors and the reviewers. Any product that may be evaluated in this article, or claim that may be made by its manufacturer, is not guaranteed or endorsed by the publisher.
